# A Structural Domain Mediates Attachment of Ethanolamine Phosphoglycerol to Eukaryotic Elongation Factor 1A in *Trypanosoma brucei*

**DOI:** 10.1371/journal.pone.0009486

**Published:** 2010-03-02

**Authors:** Eva Greganova, Manfred Heller, Peter Bütikofer

**Affiliations:** 1 Institute of Biochemistry and Molecular Medicine, University of Bern, Bern, Switzerland; 2 Mass Spectrometry and Proteomics, Department of Clinical Research, University Hospital, Bern, Switzerland; State University of Campinas (UNICAMP), Brazil

## Abstract

Ethanolamine phosphoglycerol (EPG) represents a protein modification that so far has only been found in eukaryotic elongation factor 1A (eEF1A). In mammals and plants, EPG is covalently attached to two conserved glutamate residues located in domains II and III of eEF1A. In contrast, *Trypanosoma brucei* eEF1A contains a single EPG attached to Glu362 in domain III. The sequence and/or structural requirements for covalent linkage of EPG to eEF1A have not been determined for any organism. Using a combination of biosynthetic labelling of parasites with tritiated ethanolamine and mass spectrometry analyses, we demonstrate that replacement of Glu362 in *T. brucei* eEF1A by site-directed mutagenesis prevents EPG attachment, whereas single or multiple amino acid substitutions around the attachment site are not critical. In addition, by expressing a series of eEF1A deletion mutants in *T. brucei* procyclic forms, we demonstrate that a peptide consisting of 80 amino acids of domain III of eEF1A is sufficient for EPG attachment to occur. Furthermore, EPG addition also occurs if domain III of eEF1A is fused to a soluble reporter protein. To our knowledge, this is the first report addressing amino acid sequence, or structure, requirements for EPG modification of eEF1A in any organism. Using *T. brucei* as a model organism, we show that amino acid substitutions around the modification site are not critical for EPG attachment and that a truncated version of domain III of eEF1A is sufficient to mediate EPG addition.

## Introduction

Eukaryotic elongation factor 1A (eEF1A) is one of the most abundant cytosolic proteins in all eukaryotic cells. It represents an essential component during (poly-) peptide chain elongation by binding aminoacyl-tRNAs in a GTP-dependent reaction to the acceptor site of ribosomes. After codon-anticodon match between the aminoacylated tRNA and the ribosomal acceptor site followed by GTP hydrolysis to GDP, eEF1A dissociates from the ribosome in its GDP-bound form and interacts with nucleotide exchange factor eEF1Bα, which replaces GDP by GTP to reactivate eEF1A (reviewed by [1]). Crystal structures of eEF1A in complex with components of eEF1B show that yeast eEF1A consists of three clearly distinct domains [2], [3]. Domain I harbors the GTP/GDP binding site, while binding of eEF1Bα occurs to both domains I and II [2], [4]. In addition, domain II also contains the binding site for aminoacyl-tRNAs [5]–[7], whereas in yeast domain III represents the binding site for elongation factor 3 (eEF3) [8].

Apart from its pivotal role during polypeptide synthesis, eEF1A is involved in many other cellular processes. Most notably, eEF1A binds to cytoskeletal components and modulates their interactions [9]–[11], is involved in signal transduction events [12] and mediates nuclear export of proteins [13] and mitochondrial tRNA import [14]. Interactions of eEF1A with components of the cytoskeleton, which occur independent of its translation elongation function, have been assigned to domains II and III [15], [16]. Such non-conventional roles for eEF1A may not be surprising since eEF1A amounts to 1–3% of total cytosolic protein [17] and, thus, is present in excess compared to its ligands in peptide synthesis. Since eEF1A is one of the most highly conserved proteins between eukaryotic species, it likely adopts a similar tertiary structure in all eukaryotes, as revealed by programs to predict the three-dimensional structures of proteins. Thus, the sites of interaction with its ligands are likely conserved in many other organisms as well.

The biological activity and cellular distribution of a given protein is often modulated by covalent modifications of amino acid side chains. Several protein modifications, such as phosphorylation [18], [19], lysine methylation [20], [21], and C-terminal methyl-esterification [22], have been reported to affect the activity of eEF1A during peptide synthesis in several species, yet their precise functions remain unknown (reviewed by [23]). In addition, mammalian and plant eEF1A is modified by ethanolamine phosphoglycerol (EPG) moieties attached to two conserved glutamic acid residues in domains II and III [21], [24]–[26]. The function of this modification, which so far has not been reported in any other protein except eEF1A, is completely unknown. Very recently, we have shown that eEF1A of the ancient eukaryote, *Trypanosoma brucei*, is also modified with EPG, however, in contrast to mammalian and plant eEF1A, *T. brucei* eEF1A contains a single EPG modification only. In addition, we showed that the phospholipid phosphatidylethanolamine (PE) represents the biosynthetic precursor of EPG in *T. brucei* [27]. The enzymes involved in EPG attachment to eEF1A, or the amino acid sequence or structure requirements for EPG modification of eEF1A, have not been characterized. Interestingly, *Saccharomyces cerevisiae* represents the only eukaryote so far where eEF1A has been reported not to be modified with EPG [28].

The present study was aimed at identifying sequence, or structure, motifs in *T. brucei* eEF1A necessary for EPG addition. By replacing (conserved) amino acids at, or around, the EPG modification site and by deleting individual domains of eEF1A in *T. brucei*, we found that attachment of EPG is strictly dependent on glutamate as the acceptor site and that (part of) domain III of eEF1A is sufficient to confer EPG modification, even if this domain is attached to a reporter protein.

## Methods

### Materials

Unless otherwise specified, all reagents were of analytical grade and were from Merck (Darmstadt, Germany), Sigma-Aldrich (Buchs, Switzerland) or ICN Biomedicals (Tägerig, Switzerland). DNA polymerase was obtained from Qiagen (Hombrechtikon, Switzerland). All restriction enzymes were purchased from Roche Diagnostics (Rotkreuz, Switzerland) or New England Biolabs (Ipswich, MA). [1-^3^H]ethan-1-ol-2-amine hydrochloride ([^3^H]Etn, 60 Ci mmol^−1^) was purchased from American Radiolabeled Chemicals Inc. (St. Louis, MO). Kodak MBX films were obtained from Kodak SA (Lausanne, Switzerland).

### Cell Culture and Transfection

*T. brucei* Δprocyclin#1 (EP/GPEET null mutant [29]), and the derived procyclic cell lines were cultured at 27°C in DTM [30] supplemented with 15% heat-inactivated FBS (Invitrogen, Basel, Switzerland). Trypanosomes (1×10^8^ cells) were harvested at 0.5–0.8×10^7^ cells ml^−1^ and stably transfected by electroporation with 10–15 µg of linearized plasmid DNA. Electroporation was performed with one pulse (1.5 kV charging voltage, 2.5 kV resistance, 25 µF capacitance timing, and 186 Ω resistance timing) using the BTX Electroporation 600 system (Axon Lab, Baden, Switzerland) and 0.2 cm pulse cuvette (Bio-Rad, Reinach, Switzerland). After 24 h of culture, transfectants were selected for antibiotic resistance by addition of 10 µg ml^−1^ blasticidin S HCl (Invitrogen) to the culture medium. The transfected cells were not cloned.

### Generation of Expression Vectors and Site-Directed Mutagenesis

All plasmids containing hemagglutinin-tagged eEF1A (HA-eEF1A, Table S1) were derived from the trypanosome expression vector pCorleone [31]. The open reading frame of *T. brucei* eEF1A (GeneDB Tb10.70.5670), or parts of it, were amplified from *T. brucei* genomic DNA using appropriate primer pairs (Table S2). PCR products with flanking HindIII and BamHI restriction sites were subcloned into the pCR®2.1-TOPO® vector (Invitrogen) following the manufacturer's instructions and cloned between the HindIII and BamHI sites of pCorleone. Site-specific mutations of HA-eEF1A were introduced into the parental vector pEGhaEF_(1−449)_ (Table S1) using the QuikChange® Site-Directed Mutagenesis kit (Stratagene, Basel, Switzerland) and the appropriate primers (Table S3). To generate fusion proteins containing domain III of *T. brucei* eEF1A linked to Alba 1 (HA-Alba-III) or PTP (PTP-III), the PCR product corresponding to amino acids 315–449 of eEF1A was subcloned between the EcoRI and BamHI sites of pBluescript, resulting in pBS_(315−449)_. The open reading frame of Alba 1 (GeneDB, Tb11.02.2040) was amplified from *T. brucei* genomic DNA, and a PTP-tag was generated using plasmid pN-PURO-PTP as template (GenBank, DQ172901; Schimanski, 2005) and the appropriate primers (Table S2). Resulting PCR products were inserted between the HindIII and EcoRI sites of pBS_(315−449)_. The internal BamHI restriction site in the PTP sequence was removed by site directed mutagenesis as described above (see Table S2 for primers). The resulting cassettes HA-Alba-III and PTP-III were cloned between the HindIII and BamHI sites of pCorleone, resulting in plasmids pEGhaAlba_(315−449)_ and pEGptp_(315−449)_ (Table S1). All DNA sequences were verified by sequencing (Microsynth AG, Balgach, Switzerland). Prior to transfection into *T. brucei* Δprocyclin#1, the vectors were linearized with NotI and SalI.

### In Vivo Labeling, Extraction and Immunoprecipitation

Trypanosomes during exponential growth (0.5–0.8×10^7^ cells ml^−1^) were labeled with 1 µCi ml^−1^ [^3^H]Etn for 18 h [32], [27]. Subsequently, parasites were harvested and washed twice with ice-cold TBS (10 mM Tris/HCl, pH 7.4, 144 mM NaCl) to remove non-incorporated label. Proteins from 2×10^8^ cells were extracted with 300 µl of lysis buffer (50 mM Tris, pH 7.5; 150 mM NaCl; 0.1% (v/v) Nonidet P40), containing complete protease inhibitor cocktail tablets (Roche Diagnostics), for 15 min on ice. The cell lysate was homogenized by passing three times through a 27-gauge needle and cleared by centrifugation at 16,000 g for 30 min. The supernatant was supplemented with either anti-HA Affinity Matrix (Roche Diagnostics) or IgG Sepharose 6 Fast Flow matrix (GE Healthcare, Otelfingen, Switzerland) and incubated on a rotating device overnight at 4°C. The beads were spun down and washed three times with lysis buffer. Tagged proteins bound to the matrix were released by boiling in electrophoresis sample buffer.

### SDS-Polyacrylamide Gel Electrophoresis (SDS-PAGE) and Immunoblotting

Glycine-SDS-PAGE was performed under reducing conditions using 12% polyacrylamide gels [33]. Proteins with predicted masses smaller than 15 kDa were separated by Tricine-SDS-PAGE (Schäger, 2006) in the presence of 6 M urea using 16% polyacrylamide gels. For fluorography of ^3^H-labeled proteins, gels were fixed (10% methanol, 7% acetic acid), soaked in Amplify (GE Healthcare), dried, and exposed to Kodak MBX films at −70°C. For immunoblotting, proteins were transferred at 2.5 mA/cm^2^ for 75 min onto polyvinylidene difluoride membranes (Millipore, Bedford, MA) using a semidry blotting apparatus. The blotted membranes were blocked overnight with 5% (w/v) milk powder in TBS containing 0.05% (v/v) Tween 20 and probed with primary antibody. Mouse monoclonal antibodies against HA (α-HA; Covance, Berkeley, CA) and eEF1A (α-EF; Upstate, Lake Placid, NY) were used at a dilution of 1∶3000 and 1∶5000, respectively. Primary antibodies and the PTP-tag were detected with secondary rabbit anti-mouse IgG antibody conjugated to horseradish peroxidase (Dako, Baar, Switzerland) at a dilution of 1∶5000, followed by enhanced chemiluminescence (Pierce, Lausanne, Switzerland).

### Mass Spectrometry

Immunoprecipitated HA-eEF1A was analyzed by SDS-PAGE and blotted as described above. After staining with Coomassie Brilliant Blue, proteins were cut out and exposed to on-membrane reductive alkylation and trypsin digestion [34]. The tryptic peptides were analyzed by liquid chromatography tandem mass spectrometry (nano-LC-MS/MS) using a Rheos2200 pump, equipped with GROM-SIL MB6 C8 or Magic-C18 columns (75 µm, 7 cm), coupled to an LTQ-orbitrap XL mass spectrometer. The peptide-resolving part of the nano-LC gradient was from 5% to 35% acetonitrile in 0.1% formic acid over 20 min at ∼400 nl min^−1^. The two most abundant ions were subjected to collision-induced dissociation (CID) fragmentation using a mass list comprising all expected 2+/3+ charge states of the modified and non-modified peptides. CID was triggered first in normal mode and second with the multistage activation option on when the prominent neutral loss fragment of −172 Da was observed as most abundant ion. CID spectra were recorded in the orbitrap with resolution set to 7500 at m/z 400. CID spectra were interpreted by Phenyx software (Genebio SA, Geneva, Switzerland) using carboamidomethylation of cysteine as a fixed modification, and oxidation of methionine and EPG on glutamic acid as variable modification. All nano-LC-MS/MS chromatograms were manually controlled for the presence of the expected peptides.

## Results

### Amino Acid Sequence Requirement for EPG Attachment

**Figure 1:**
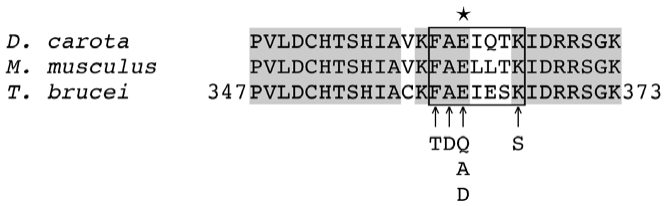
Amino acid sequence comparison around the EPG modification site. The amino acid sequence of *T. brucei* eEF1A between residues 347 and 373 is compared to the corresponding eEF1A sequences from *Daucus carota* (GenBank, BAA02205) and *Mus musculus* (GenBank, NP_034236) using ClustalW. A grey background indicates amino acid identity between the sequences. The arrows in indicate the conserved residues at, or around, the EPG modification site of *T. brucei* eEF1A (Glu362) that were mutated in this study (E362Q, E362A, E362D, F360T, A361D, K366S). The box marks the tryptic fragments containing the EPG modification site (indicated by the asterisk).

eEF1A shows a very high amino acid sequence identity over the entire polypeptide chain between different organisms. Interestingly, however, the residues around the EPG modification site, Glu362 in *T. brucei* [27], are less well conserved (Fig. 1). To study the importance of conserved amino acids within this stretch of the primary sequence, i.e. between Cys358 and Ile367, for EPG attachment to eEF1A, we introduced point mutations at, or around, Glu362 in ectopically expressed, HA-tagged full-length eEF1A (HA-eEF1A) in *T. brucei* procyclic forms. Attachment of EPG to eEF1A was monitored by incubating trypanosomes in culture with [^3^H]Etn, which has been used before to label eEF1A in mammalian cells [24], [25], plants [26], and *T. brucei* [27]. Since this procedure also results in labeling of abundant GPI-anchored surface proteins in trypanosomes (see, e.g., [35], [36], [32], [37]), we chose the *T. brucei* procyclic cell line, Δprocyclin#1, which lacks the expression of the major GPI-anchored proteins, EP and GPEET procyclins [29], for all transfection experiments. Using this cell line, labeling with [^3^H]Etn results in a single radioactive band at 49 kDa after SDS-PAGE and fluorography, allowing easy identification of wild-type or HA-tagged eEF1A ([27]; see also below). Expression of the various HA-tagged forms eEF1A had no effect on growth of the parasites in culture.

**Figure 2:**
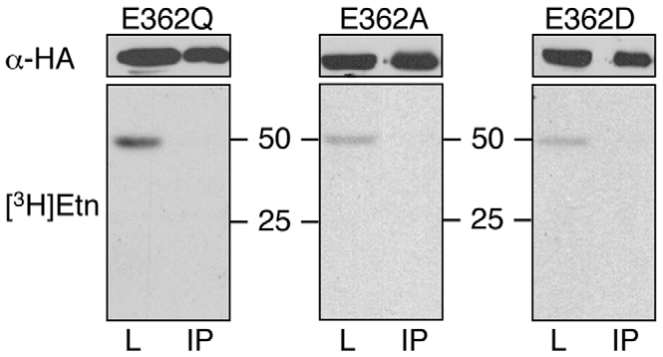
Expression of mutated HA-eEF1A and labeling with [^3^H]Etn. *T. brucei* Δprocyclin#1 expressing HA-eEF1A carrying the point mutations E362Q, E362A or E362D were incubated in the presence of [^3^H]Etn for 18 h. HA-eEF1A in the cell lysates (L) and after immunoprecipitation (IP) were analyzed by SDS-PAGE using 12% acrylamide gels, followed by immunoblotting using α-HA antibody (upper panels), or fluorography (lower panels). The lanes contain material from 1×10^7^ parasites, except for the lanes with the immunoprecipitates in the fluorographs, which contain material prepared from 1.8×10^8^ cell equivalents.

Immunoblotting experiments showed that HA-eEF1A proteins carrying the point mutations E362Q, E362A and E362D were expressed in *T. brucei* procyclic forms and could be immunoprecipitated using α-HA antibodies (Fig. 2, upper panels). As expected, replacement of the glutamate residue representing the EPG attachment site by glutamine (E362Q), or alanine (E362A), resulted in complete loss of [^3^H]Etn labeling of HA-eEF1A (Fig. 2, lower left and middle panels). In addition, substitution of glutamate by aspartate (E362D) also completely abolished labeling of HA-eEF1A (Fig. 2, lower right panel), indicating that the length of the amino acid side chain is critical for [^3^H]Etn incorporation into eEF1A. The radioactive bands at 49 kDa in the lysates of the respective experiments represent endogenous [^3^H]Etn-labeled eEF1A, demonstrating that uptake and incorporation of [^3^H]Etn into wild-type eEF1A was unaltered in these mutant parasites. To study the importance of the three conserved amino acids in the vicinity of the EPG attachment site, Phe360, Ala361 and Lys366, we expressed HA-eEF1A proteins carrying the point mutations F360T, A361D or K366S in trypanosomes. The results after incubating parasites expressing these proteins with [^3^H]Etn show that the mutations didn't prevent labeling of HA-eEF1A with [^3^H]Etn (results not shown).

**Figure 3:**
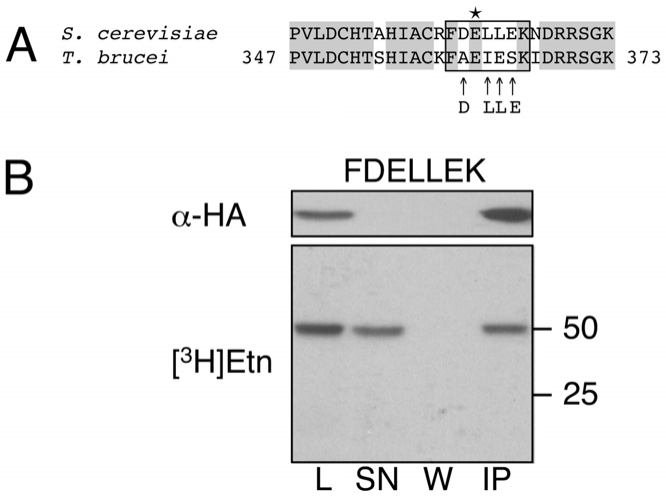
Replacement of a *T. brucei* eEF1A peptide sequence by its yeast homologue. (A) The primary sequence of *T. brucei* eEF1A between residues 347 and 373 is aligned to the corresponding eEF1A sequence from *S. cerevisiae* (GeneDB, YBR118W) using ClustalW (http://clustalw.genome.ad.jp). A grey background indicates amino acid identity between the sequences. The arrows indicate the residues that were sequentially mutated in *T. brucei* eEF1A (A361D, I363L, E364L, S365E) to substitute the stretch between Phe360 and K366 (FAEIESK) by the corresponding *S. cerevisiae* sequence (FDELLEK). The asterisk indicates the EPG modification site and the frame marks the tryptic fragments analyzed by mass spectrometry. (B) *T. brucei* Δprocyclin#1 expressing HA-eEF1A, containing the yeast sequence FDELLEK instead of FAEIESK, were incubated with [^3^H]Etn for 18 h. Proteins in the cell lysate (L), supernatant (SN), wash solution (W) and immunoprecipitate (IP) were separated and analyzed by immunoblotting as described in Fig. 2. The lanes contain material from 1×10^7^ parasites, except for the lanes with the immunoprecipitates in the fluorographs, which contain material prepared from 1.8×10^8^ cell equivalents.

Since the conserved glutamate residue in eEF1A from *S. cerevisiae* is not modified with EPG [28], we converted the tryptic peptide of *T. brucei* eEF1A, FAEIESK, carrying the EPG modification site, to the corresponding tryptic peptide from yeast eEF1A, FDELLEK (Fig. 3A), and studied its potential modification in *T. brucei*. Our results show that replacement of all four amino acids around the EPG attachment site to mimic the yeast sequence had no effect on [^3^H]Etn labeling of HA-eEF1A in *T. brucei* (Fig. 3B).

### Mass Spectrometry Analysis of EPG Attachment to Mutated HA-eEF1A

**Figure 4:**
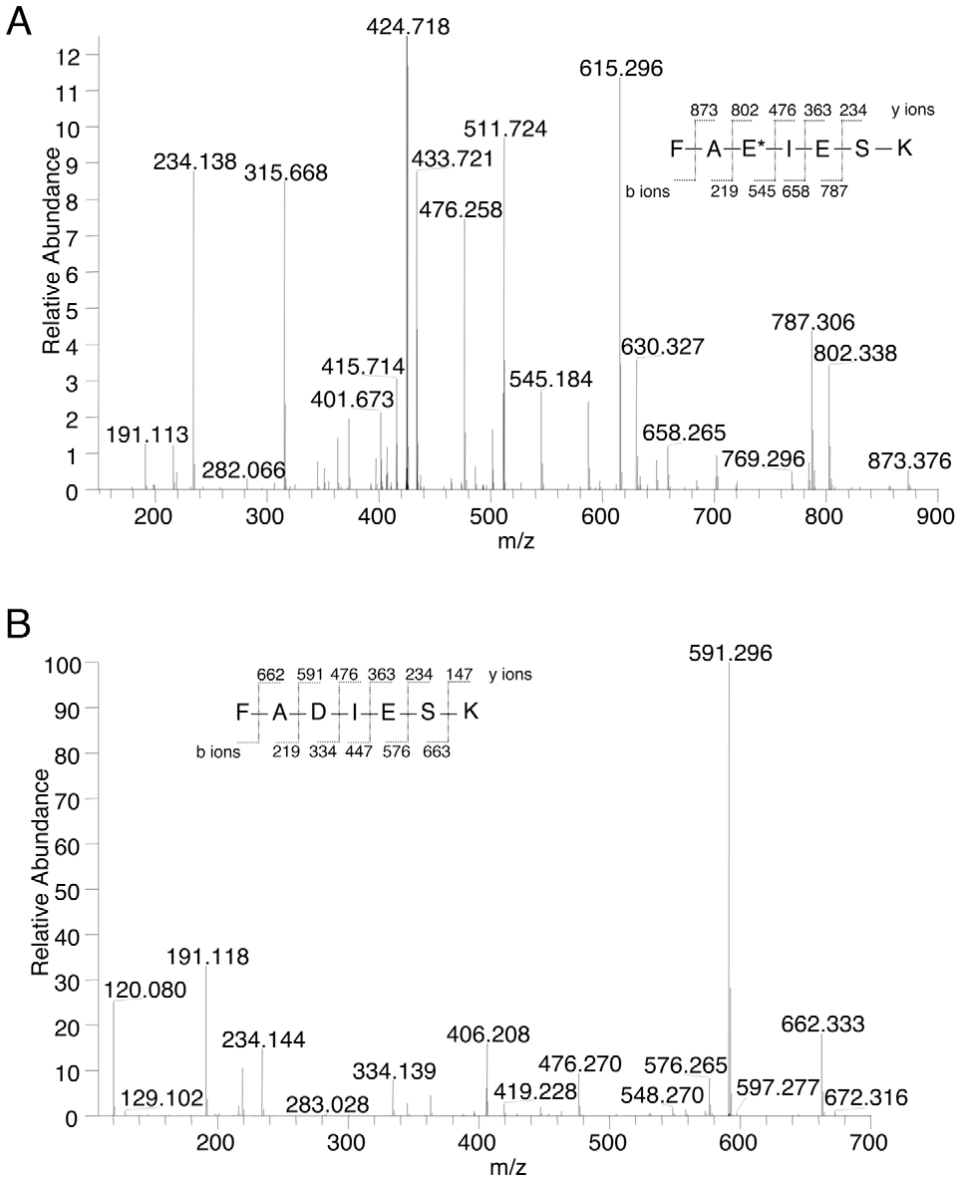
Analysis of EPG attachment by mass spectrometry. Tryptic peptides from *T. brucei* HA-eEF1A mutants were subjected to nano-LC-MS/MS and the collision-induced fragmentation spectra of ions *m/z* 510.736 and 405.206, corresponding to EPG-modified FAE*IESK and unmodified FADIESK, respectively, are shown. The intensity scale in panel A is set to 12.5% of the most abundant ion of 424.718, which corresponds to the doubly charged parent ion after neutral loss of glycerol phosphate. The y and b type ions are marked with the corresponding *m/z* of the singly charged ions given above or underneath the amino acid sequence.

Although incorporation of [^3^H]Etn into eEF1A is a helpful and convenient marker for EPG labeling, it provides no information on the exact site of modification, or if the entire EPG moiety–and not only ethanolamine (see [38]) – is attached. To obtain this information, purified (immunoprecipitated) HA-tagged proteins were reduced, alkylated, digested with trypsin, and analyzed by nano-LC-MS/MS. All tryptic peptides recovered matched the deduced amino acid sequence of wild-type *T. brucei* eEF1A (results not shown), except for the peptides carrying the point mutations (see below). HA-tagged wild-type eEF1A revealed a tryptic fragment with *m/z* 1020.465 and 510.736, representing the [M+H]^+^ and [M+2H]^2+^ ions of the heptapeptide FAEIESK (Phe360 to Lys366, 823.420 Da), carrying an EPG moiety with the predicted mass of 197.045 Da (results not shown; see also Table 1 and [27]). MS/MS analysis of the parental ion at *m/z* 510.736 identified Glu362 in the sequence of the tryptic peptide FAE*IESK (with E* indicating EPG-modified glu) as the site of EPG attachment (Fig. 4A). The ion spectrum showed a major [M+2H]^2+^ ion of *m/z* 424.718 after neutral loss of 172.014 Da due to elimination of glycerol phosphate, leaving Glu362 modified with NH–CH = CH_2_. In contrast, analysis of the HA-eEF1A E362D mutant revealed a tryptic fragment with *m/z* 809.404 and 405.206, representing the [M+H]^+^ and [M+2H]^2+^ ions of the unmodified heptapeptide FADIESK (results not shown; see also Table 1), with MS/MS analysis of the parental ion at *m/z* 405.206 confirming the absence of EPG (Fig. 4B). Similarly, only the unmodified tryptic peptides FAQIESK and FAAIESK were detected in the E362Q and E362A mutants, respectively (Table 1). In contrast, point mutations at the conserved residues Ala361 and Lys366 had no effect on EPG attachment to *T. brucei* HA-eEF1A, as demonstrated by detection of the EPG-modified peptides FDE*IESK, FAE*IESSIDR and FAE*IESSIDRR (Table 1). For unknown reasons, no TAE*IESK peptide of the F360T mutant could be detected by MS analysis; however, the successful introduction of the point mutation in the open reading frame of HA-eEF1A was confirmed by PCR and DNA-sequencing. Finally, MS analysis of HA-eEF1A mimicking the yeast eEF1A sequence around the EPG modification site showed the presence of the EPG-modified peptide, FDE*LLEK (Table 1).

**Table 1:** Characteristic ions of the tryptic fragments of *T. brucei* eEF1A proteins detected by mass spectrometry.

Protein	Tryptic fragment	[M+H]^+^	[M+H]^2+^	EPG
Wild-type	FAE*IESK/FAEIESK	1020.465/823.420^a^	510.736/412.214^a^	+
E362Q	FAQ*IESK/FAQIESK	n.d./822.435	n.d./411.722	
E362A	FAA*IESK/FAAIESK	n.d./765.414	n.d./383.210	
E362D	FAD*IESK/FADIESK	n.d./809.404	n.d./405.206	
F360T	TAE*IESK/TAEIESK	o	o	
A361D	FDE*IESK/FDEIESK	1064.454/867.409	532.731/434.209	+
K366S	FAE*IESSIDR/FAEIESSIDR &	1363.613/n.d.	682.313/n.d.	+
	FAE*IESSIDRR/FAEIESSIDRR	1519.714/n.d.	760.362/n.d.	+
A361D/I363L	FDE*LESK/FDELESK	1064.444/867.401	532.725/434.204	+
A361D/I363L/E364L	FDE*LLSK/FDELLSK	1048.485/851.442	524.745/426.225	+
A361D/I363L/E364L/S365E	FDE*LLEK/FDELLEK	1090.491/893.447	545.749/447.228	+
315–394	FAE*IESK/FAEIESK	1020.466/823.432	510.736/412.219	+
348–449	FAE*IESK/FAEIESK	1020.463/823.420	510.736/412.213	+
HA-Alba-III	FAE*IESK/FAEIESK	1020.466/823.419	510.736/412.214	+
PTP-III	FAE*IESK/FAEIESK	1020.463/n.d.	510.734/412.212	+

Purified *T. brucei* HA-eEF1A was digested with trypsin and subjected to nano-LC-MS/MS as described in Experimental Procedures. Tryptic fragments containing the site of potential EPG attachment (marked by the asterisk) with the corresponding [M+H]^+^and [M+2H]^2+^ ions are listed.

n.d., not detected

o, no fragments detected

a the relative intensities of the [M+H]^+^ ions of the EPG-modified (*m/z* 1020.465) and unmodified (*m/z* 823.420) tryptic peptides suggest that >95% of wild-type eEF1A is modified with EPG.

The last column indicates the presence (+) of the EPG modification based on the ion data.

MS analysis of all immunoprecipitated HA-eEF1A proteins showed the presence of trace amounts of the tryptic peptide FAE*IESK (results not shown), indicating that HA-eEF1A interacts with endogenous eEF1A. This is consistent with reports showing that eEF1A in *Tetrahymena* exists as homodimers and that dimer formation occurs via domain III [11], [39].

Together, these results demonstrate that the attachment of EPG to eEF1A only occurs if Glu362 is strictly conserved, but is not affected by substitution of conserved amino acids in the vicinity of the modification site. Based on these observations, and our failure to identify an amino consensus sequence in eEF1A that could mediate EPG attachment, we investigated if a structural domain may determine EPG modification of eEF1A.

### EPG Attachment to eEF1A Deletion Mutants

**Figure 5:**
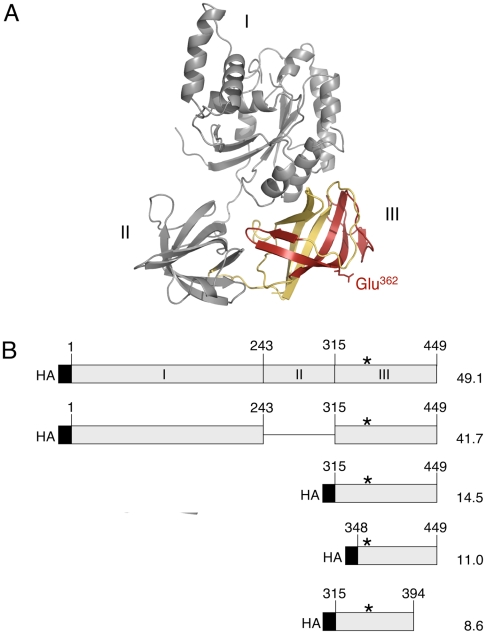
Tertiary structure of *T. brucei* eEF1A and design of deletion mutants. (A) Predicted three-dimensional structure of *T. brucei* eEF1A using PyMol (http://www.pymol.org). The site of EPG attachment, Glu362, is exposed on the surface of domain III. Domain III is highlighted in colour; the amino acid sequence common to the minimal structural units showing EPG attachment (residues 348–394) is indicated in red, the flanking N- and C-terminal stretches (residues 315–347 and 395–449, respectively) are in yellow. (B) Overview of HA-tagged deletion mutants of *T. brucei* eEF1A. The numbers indicate the positions of the amino acids in the full length protein. The black boxes represent the HA-tags and the asterisks indicate the EPG modification site (Glu 362). Predicted molecular masses are indicated in kDa on the right.

**Figure 6:**
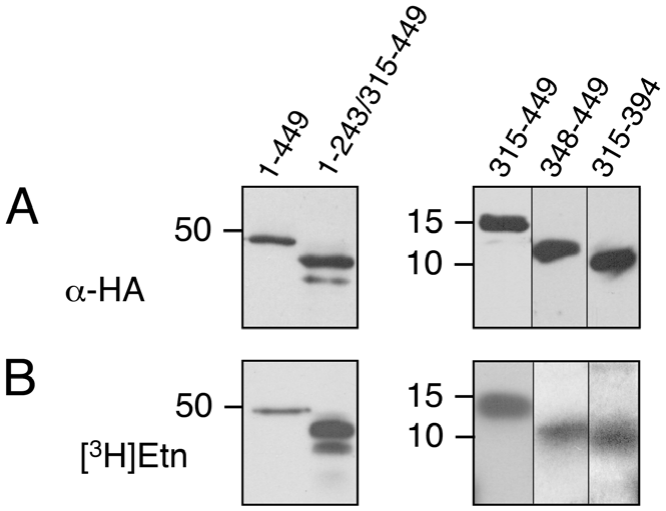
Expression of HA-eEF1A deletion mutants and labeling with [^3^H]Etn. *T. brucei* Δprocyclin#1 expressing full length HA-eEF1A, or HA-tagged deletion mutants, were incubated in the presence of [^3^H]Etn for 18 h. Subsequently, parasites were lysed, and HA-tagged proteins were immunoprecipitated and analyzed by glycine-SDS-PAGE (left panels) or Tricine-SDS-PAGE (right panels). HA-eEF1A was visualized by immunoblotting using α-HA (upper panels), whereas labeling with [^3^H]Etn was analyzed by fluorography (lower panels). The lanes contain material from 1×10^7^ parasites, except for the lanes with the immunoprecipitates in the fluorographs, which contain material prepared from 1.8×10^8^ cell equivalents. The deletion mutants are marked according to Fig. 5B. Molecular mass markers are indicated in kDa.

The predicted three dimensional structure of *T. brucei* eEF1A shows three distinct structural domains (Fig. 5A, termed I, II and III) and closely matches the three dimensional structure of *S. cerevisiae* eEF1A as determined by X-ray crystallography [3]. The Glu362 residue modified by EPG in *T. brucei* eEF1A is located in a predicted β-sheet in domain III (Fig. 5A). To determine the structural requirements for the attachment of EPG to Glu362, several HA-tagged deletion mutants of *T. brucei* eEF1A were designed (Fig. 5B) and transfected into *T. brucei* procyclic forms. Expression of HA-eEF1A and incorporation of [^3^H]Etn was monitored as described for the point mutations. Immunoblotting experiments using α-HA demonstrate that eEF1A proteins lacking domain II (construct 1–243/315–449) or domains I + II (construct 315–449), as well as HA-tagged full-length eEF1A (construct 1–449), were expressed (Fig. 6A). The minor band migrating below HA-tagged eEF1A lacking domain II likely represents a proteolytic degradation product of the protein construct. Interestingly, after incubation of trypanosomes with [^3^H]Etn, the two truncated proteins were strongly labeled (Fig. 6B), indicating that domains I and II are not required for incorporation of [^3^H]Etn into domain III of *T. brucei* eEF1A.

To identify a minimal structural unit of domain III needed for [^3^H]Etn incorporation, we generated deletion mutants of domain III and analyzed their expression and labeling with [^3^H]Etn. The results show that the truncated forms of domain III, after deletion of 33 amino acids at the N-terminus (construct 348–449) or 55 amino acids at the C-terminus (construct 315–394), were expressed (Fig. 6A) and, in addition, labeled with [^3^H]Etn (Fig. 6B). In contrast, no expression was observed after truncating both the N- and the C-terminus of domain III, resulting in a peptide fragment consisting of amino acids 348–394 of eEF1A (results not shown). To confirm the site of attachment and the structure of the modification, the deletion mutants were analyzed by nano-LC-MS/MS. The results showed that the purified (immunoprecipitated) HA-tagged eEF1A peptides 348-449 and 315–394 were modified at Glu362 and that the entire EPG structure was present (Table 1). Together, these results demonstrate that a peptide consisting of 80 amino acids of domain III of eEF1A is sufficient for EPG modification in *T. brucei*.

### EPG Attachment to Chimeric Proteins

**Figure 7:**
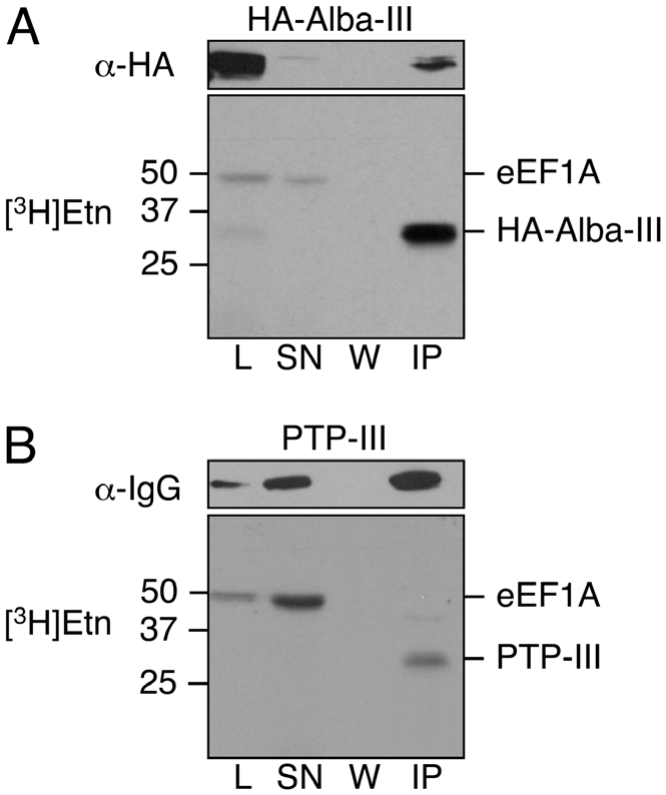
Expression of domain III fusion mutants and labeling with [^3^H]Etn. (A), (B), *T. brucei* Δprocyclin#1 expressing HA-Alba-III and PTP-III fusion proteins were labeled with [^3^H]Etn for 18 h. Proteins in the cell lysates (L), supernatants (SN), wash solutions (W) and immunoprecipitates (IP) were analyzed by SDS-PAGE using 12% acrylamide gels. SDS PAGE was followed either by immunoblotting (upper panels) or fluorography (lower panels). For immunoblotting α-HA antibody or α-IgG were used to detect HA-Alba-III or PTP-III, respectively. The lanes contain material from 1×10^7^ parasites, except for the lanes with the immunoprecipitates in the fluorographs, which contain material prepared from 1.8×10^8^ cell equivalents.

Having demonstrated that domain III of eEF1A alone is modified by EPG, we next studied if linking this domain to other proteins confers EPG modification to the chimeric proteins. For this purpose, we linked domain III of eEF1A to the small cytosolic protein, *T. brucei* Alba 1, or to the tandem affinity purification tag, PTP [40], resulting in the chimeric proteins HA-Alba-III or PTP-III with predicted molecular masses of 30 or 33 kDa, respectively. Immunoblotting experiments showed that both constructs were expressed in *T. brucei* procyclic forms (Fig. 7A, B, upper panels) and incorporated [^3^H]Etn (Fig. 7A, B, lower panels). Nano-LC-MS/MS analysis of immunoprecipitated proteins confirmed the presence of the entire EPG moiety attached to Glu362 of domain III (Table 1). These data demonstrate that domain III of eEF1A is modified with EPG as independent structural unit, regardless if it is expressed as individual polypeptide or attached to other proteins.

## Discussion

Based on the published three-dimensional structure of yeast eEF1A [3], [4] and the high amino acid sequence homology between eEF1A from different organisms, the EPG modification site in *T. brucei* eEF1A, Glu362, is predicted to be on the surface of a β-sheet in domain III. Using *in vivo* labeling with [^3^H]ethanolamine and mass spectrometric analyses, we now showed that a peptide consisting of amino acids 315–394 of the primary sequence, which is located in domain III of eEF1A, is sufficient for EPG attachment to occur. This was demonstrated by expressing HA-tagged forms of eEF1A lacking domains I and/or II, or the N- and C-terminal parts of domain III. Similarly, EPG modification also occurred on chimeric proteins consisting of domain III of *T. brucei* eEF1A fused to soluble reporter proteins, demonstrating that domain III contains all necessary information to become EPG-modified. In addition, point mutations of conserved amino acids around the EPG attachment site didn't prevent EPG modification, indicating that a specific primary sequence does not appear to be involved in EPG attachment. Furthermore, while replacement of Glu362 by Ala or Gln was expected to block EPG attachment, replacement by Asp also completely abolished EPG addition, demonstrating that the enzyme catalyzing attachment of EPG, or its precursor molecule, is highly specific for Glu at this position and doesn't recognize a carboxyl group linked to a shorter amino acid side chain.

Mammalian and plant eEF1A contain two EPG residues attached to Glu in domains II and III [24]–[26]. This is in contrast to *T. brucei*, where eEF1A is modified with a single EPG moiety in domain III only ([27] and this report), despite the fact that the second potential modification site, Glu289, is strictly conserved between trypanosomes, mammals and plants. Our results demonstrate that this residue remains unmodified in *T. brucei* even if EPG attachment to Glu362 is prevented by point mutations.

Until today, EPG attachment has only been described in eEF1A. Two other amino acid side chain modifications show a similar selectivity for single proteins. In analogy to eEF1A, these proteins, eukaryotic translation initiation factor 5A (eIF5A) and elongation factor 2 (eEF2), are also involved in polypeptide elongation. First, hypusine (Nε-(4-amino-2-hydroxybutyl)-lysine) represents a post-translationally modified lysine residue in eIF5A ([41]; reviewed by [42]). It has been described in eukaryotes and archaeabacteria, but is absent from bacteria. Recent reports have demonstrated that eIF5A associates in a hypusine-dependent way with actively translating ribosomes, suggesting that eIF5A may have a role in polypeptide elongation rather than, or in addition to, translation initiation [43]–[45]. In addition, the hypusine modification may be involved in correct localization of eIF5A within the cytosol [46]. The biosynthetic pathway leading to hypusine formation has been established (reviewed by [42]). Furthermore, a minimal structural domain of eIF5A necessary for hypusine modification has been identified [47].

Second, diphthamide, 2-(3-carboxyamido-3-(trimethylammonio)propyl)-histidine represents a post-translationally modified histidine residue found only in eEF2 ([48]; reviewed by [49]). Similar to hypusine, diphthamide has been conserved throughout eukaryotic and Archaea evolution. It represents the target for ADP-ribosylation by bacterial toxins, rendering eEF2 inactive and causing inhibition of protein synthesis. However, its physiological role is still unclear, as several studies have demonstrated that eEF2 function is not directly dependent on diphthamide formation (reviewed by [49]). More recently, evidence for a direct role of diphthamide in cell function has been obtained when yeast strains expressing diphthamide-deficient eEF2 were shown to have increased -1 frame-shifting [50]. In addition, diphthamide was demonstrated to protect mutant mammalian cells from the action of the ribosome-inactivating protein, ricin [51]. The biosynthetic pathway leading to attachment of diphthamide to eEF2 has been completely solved in *S. cerevisiae* (reviewed by [49]). In contrast to EPG addition to eEF1A, the conserved amino acid residues around the modification site are critical for diphthamide formation in eEF2 [50], [52].

Similar to hypusine and diphthamide, EPG is absent in bacteria [25]. However, whereas hypusine and diphthamide seem to be present in all eukaryotes, EPG was reported to be absent in eEF1A from *S. cerevisiae* [28]. In line with this observation, we were unable to label yeast eEF1A using [^3^H]ethanolamine *in vivo* (results not shown). To test if yeast eEF1A lacks EPG because the biosynthetic pathway is deficient or if structure or sequence differences in yeast eEF1A prevent EPG attachment, we tried to express a tagged form of *T. brucei* eEF1A in *S. cerevisiae*, and *vice versa*. Unfortunately, despite several attempts we were unable to obtain expression of HA-tagged *T. brucei* eEF1A in different yeast strains. In contrast, HA-tagged eEF1A from *S. cerevisiae* was readily expressed in *T. brucei* procyclic forms, however, it was not modified with [^3^H]ethanolamine (results not shown). At present, it is unclear if yeast eEF1A was not modified in *T. brucei* because it is not properly folded. However, our results showing that replacement of the primary sequence around the EPG attachment site of *T. brucei* eEF1A by the yeast sequence didn't affect EPG attachment in *T. brucei*, demonstrate that the different amino acid sequence of yeast eEF1A *per se* is not responsible for the lack of EPG modification in *S. cerevisiae*.

The pathways involved in EPG attachment to eEF1A, and its biological functions, are completely unknown. Our observations that domain III of eEF1A, expressed either as separate entity or fused to tagged proteins in *T. brucei*, is modified with EPG suggest that it may be feasible to identify interaction partners of EPG-modified eEF1A using tagged domain III to advance the general knowledge on the synthesis and role of this enigmatic protein modification using trypanosomes as model system.

## Supporting Information

Table S1 (0.05 MB DOC)

Table S2 (0.06 MB DOC)

Table S3 (0.07 MB DOC)
