# Supplementary material for: A Structural Domain Mediates Attachment of Ethanolamine Phosphoglycerol to Eukaryotic Elongation Factor 1A in Trypanosoma brucei
Source: PLoS One. 2010 Mar 2;5(3):e9486. doi: 10.1371/journal.pone.0009486 (PMC2830473; doi:10.1371/journal.pone.0009486)
Supplement: Table S1 — (0.05 MB DOC) [file pone.0009486.s001.doc]

Table S1: Plasmids used for transfection of *T. brucei* Δprocyclin#1

| Point mutants | Deletion mutants | Fusion mutants |
| --- | --- | --- |
| pEGhaEF_(E362Q)_ | pEGhaEF_(1-449)_ | pBS_(315-449)_ |
| pEGhaEF_(E362A)_ | pEGhaEF_(1-243/315-449)_ | pEGhaAlba_(315-449)_ |
| pEGhaEF_(E362D)_ | pEGhaEF_(315-449)_ | pEGptp_(315-449)_ |
| pEGhaEF_(F360T)_ | pEGhaEF_(348-449)_ |  |
| pEGhaEF_(A361D)_ | pEGhaEF_(315-394)_ |  |
| pEGhaEF_(I363L)_ |  |  |
| pEGhaEF_(E364L)_ |  |  |
| pEGhaEF_(S365E)_ |  |  |
| pEGhaEF_(K366S)_ |  |  |
