# Supplementary material for: A Structural Domain Mediates Attachment of Ethanolamine Phosphoglycerol to Eukaryotic Elongation Factor 1A in Trypanosoma brucei
Source: PLoS One. 2010 Mar 2;5(3):e9486. doi: 10.1371/journal.pone.0009486 (PMC2830473; doi:10.1371/journal.pone.0009486)
Supplement: Table S2 — (0.06 MB DOC) [file pone.0009486.s002.doc]

Table S2: Primers used for generation of *T. brucei* eEF1A deletion and fusion mutants

| Primer | Direction | Sequence (5`🡪3`) |
| --- | --- | --- |
| Tb5670_1 | forward | TA*AAGCTT*ATG**TACCCTTATGACGTACCAGACTATGCA**ATGGGAAAGGAAAAGGTG |
| Tb5670_243 | reverse | GTCGTTCTTGGTGTTACCCTTGTACACGTCCTGCAG |
| Tb5670_315/a | forward | AAGGGTAACACCAAGAACGAC |
| Tb5670_315/b | forward | TA*AAGCTT*ATG**TACCCTTATGACGTACCAGACTATGCA**GGTAACACCAAGAACGAC |
| Tb5670_449 | reverse | CG*GGATCC*TTATTTCTTCGAAGCCTT |
| Tb5670_348 | forward | TA*AAGCTT*ATG**TACCCTTATGACGTACCAGACTATGCA**GTGCTGGACTGCCACACA |
| Tb5670_394 | reverse | TA*GGATCC*TTACGGCACCATGCGCA |
|  |  |  |
| PTP | forward | GAGCCC*AAGCTT*ATGGCAGGCCTTGCGCA |
| PTP | reverse | CTCCTC*GAATTC*TTTCCCATCAATAAGACG |
| PTP V151V | forward | CTAGAAGATCAGGTAGATCCTCGTCTTATT |
| PTP V151V | reverse | AATAAGACGAGGATCTACCTGATCTTCTAG |
| Tb2040 | forward | GAGCCC*AAGCTT*ATG**TACCCTTATGACGTACCAGACTATGCA**ATGACGACAGGAAAGAGC |
| Tb2040 | reverse | CTCCTC*GAATTC*CGCTGTAGCCTTCTCCTT |
| Tb5670_315/c | forward | GAGGAG*GAATTC*GGTAACACCAAGAACGAC |

Nucleotides in italics indicate the restriction sites for HindIII, BamHI or EcoRI; underlined nucleotides mark mutated triplets and

nucleotides in bold correspond to the HA tag
