# Supplementary material for: A Structural Domain Mediates Attachment of Ethanolamine Phosphoglycerol to Eukaryotic Elongation Factor 1A in Trypanosoma brucei
Source: PLoS One. 2010 Mar 2;5(3):e9486. doi: 10.1371/journal.pone.0009486 (PMC2830473; doi:10.1371/journal.pone.0009486)
Supplement: Table S3 — (0.07 MB DOC) [file pone.0009486.s003.doc]

Table S3: Primers used to introduce point mutations into *T. brucei* HA-eEF1A

| Primer | Direction | Sequence (5`🡪3`) |
| --- | --- | --- |
| 362Q | forward | GCAAGTTCGCGCAGATCGAGTCGAAGA |
| 362Q | reverse | TCTTCGACTCGATCTGCGCGAACTTGC |
| 362A | forward | TGCAAGTTCGCGGCGATCGAGTCGAAG |
| 362A | reverse | CTTCGACTCGATCGCCGCGAACTTGCA |
| 362D | forward | TGCAAGTTCGCGGACATCGAGTCGAAG |
| 362D | reverse | CTTCGACTCGATGTCCGCGAACTTGCA |
| 360T | forward | ATTGCCTGCAAGACCGCGGAGATCGAG |
| 360T | reverse | CTCGATCTCCGCGGTCTTGCAGGCAAT |
| 361D | forward | GCCTGCAAGTTCGACGAGATCGAGTCG |
| 361D | reverse | CGACTCGATCTCGTCGAACTTGCAGGC |
| 363L | forward | AAGTTCGACGAGCTCGAGTCGAAGATC |
| 363L | reverse | GATCTTCGACTCGAGCTCGTCGAACTT |
| 364L | forward | TTCGACGAGCTCCTGTCGAAGATCGAC |
| 364L | reverse | GTCGATCTTCGACAGGAGCTCGTCGAA |
| 365E | forward | GACGAGCTCCTGGAGAAGATCGACCGT |
| 365E | reverse | ACGGTCGATCTTCTCCAGGAGCTCGTC |
| 366S | forward | GAGATCGAGTCGAGTATCGACCGTCGC |
| 366S | reverse | GCGACGGTCGATACTCGACTCGATCTC |

Underlined nucleotides indicate mutated triplets
